# A complete cross-over design evaluating canine acceptance of Carprieve® and Rimadyl® carprofen chewable tablets in healthy dogs

**DOI:** 10.1186/s12917-019-2124-1

**Published:** 2019-11-05

**Authors:** Diana M. A. Dewsbury, Keith D. DeDonder, Darrell J. Rezac, Natalia Cernicchiaro

**Affiliations:** 1 Center for Outcomes Research and Epidemiology and Department of Diagnostic Medicine / Pathobiology, College of Veterinary Medicine, Kansas State University, 1620 Denison Avenue, Manhattan, KS 66506 USA; 2 Veterinary and Biomedical Research Center Inc., 9027 Green Valley Drive, Manhattan, KS 66502 USA

**Keywords:** Canine, Carprofen, Carprieve, Cross-over, Dog, Osteoarthritis, Rimadyl

## Abstract

**Background:**

Osteoarthritis (OA) affects nearly 20% of all dogs greater than one year of age. Clinical signs include pain, discomfort, lameness, and ultimately lead to disability. Although there is currently no known cure, there are many therapeutic options that can slow the progression and alleviate the associated signs. There is ample supportive evidence demonstrating the efficaciousness of carprofen, a non-steroidal anti-inflammatory drug, in managing signs of OA. Since the approval of the pioneer product (Rimadyl®, Zoetis; Kalamazoo, Michigan), the United States Food and Drug Administration (FDA) has assented to several other generic, bioequivalent products. The objective of this 2 × 2 complete cross-over design was to assess the acceptance of two bioequivalent carprofen liver-flavored chewable tablets (containing 25 mg carprofen), Rimadyl® and Carprieve® (Norbrook Laboratories Limited; Newry, Northern Ireland) in 37 healthy purpose-bred dogs.

**Results:**

Overall, 73.0% (27/37) and 70.3% (26/37) of dogs voluntarily accepted Rimadyl® and Carprieve®, respectively. Considering acceptability tests paired by individual dog, 64.9% of dogs (*n* = 24) voluntarily accepted both Rimadyl® and Carprieve® chewable tablets whereas 21.6% (8) of dogs denied or partially accepted both products offered. Three dogs (8.1%) fully accepted Rimadyl® but did not accept Carprieve®. Conversely, two dogs (5.4%) fully accepted Carprieve® but did not accept Rimadyl®. Canine acceptability did not significantly differ between Carprieve® and Rimadyl® carprofen chewable tablets (*P* = 0.65).

**Conclusions:**

Utilizing a 2 × 2 complete cross-over design, this study provides evidence that canine acceptability of a single-dose did not differ between Carprieve® and Rimadyl® chewable tablets.

## Background

Osteoarthritis (OA) is a complex syndrome that has been reported to affect approximately 20% of dogs over the age of one [1]. Clinical signs primarily include pain and discomfort which worsen over time ultimately resulting in lameness and disability [1]. Although there are currently no known cures, there are many treatments available to manage signs in dogs, including but not limited to non-steroidal anti-inflammatory drugs (NSAIDs), analgesics, nutraceuticals, functional foods, physical therapy, alternative therapies (e.g., stretching, acupuncture), and elective surgeries to slow progression or replace the joint entirely [2, 3]. A systematic review synthesizing literature on therapeutic treatments for canine OA found that NSAIDs, including carprofen, firocoxib, and meloxicam, effectively managed the symptoms associated with OA [2]. Most of the published literature pertained to studies evaluating Rimadyl® (Zoetis; Kalamazoo, Michigan), the pioneer carprofen product [4–9]. In addition to Rimadyl®, the United States Food and Drug Administration (FDA) has approved several bioequivalent, generic carprofen products for commercial use [10].

Although the pharmacokinetics are considered bioequivalent between generic products and Rimadyl®, acceptance of the product and pet owner compliance to the treatment protocol, also crucial to drug efficacy, are not guaranteed [11]. Pet acceptability facilitates convenience of treatment administration and protocol compliance by the pet owner [12]. Veterinary drug products, including carprofen, come in a variety of presentations including, but not limited to: tablets, caplets, chewable tablets, and injectable solutions. Whereas treats exist to house non-chewable formulations or ease treatment administration to dogs resisting oral medication, those products add additional costs for the pet owner and may contribute to known causes of arthritis such as obesity. Developing highly palatable formulations, measured in terms of acceptance and preference, are at the forefront of pet food and orally administered veterinary drug product development. Canine acceptability, notably voluntary consumption, is especially important with medications that are administered daily for long periods of time for chronic conditions, such as OA [12]. Similarly, costs associated with veterinary care influence pet owner compliance to veterinary prescribed treatment protocols and ultimately, the quality of life of the pet. One in five pet owners admitted to taking one of these cost-cutting steps, 1) delayed purchasing of prescribed prescriptions, 2) used a less than recommended prescription dose, or 3) declined purchasing a medication their pet was prescribed altogether [13].

Carprieve® (25 mg carprofen; Norbrook Laboratories Limited; Newry, Northern Ireland) chewable tablets are an approved generic of Rimadyl® chewable tablets to treat symptoms associated with OA and manage pain following surgery in dogs. While the safety, efficacy, and bioequivalence of the carprofen products were demonstrated prior to receiving initial FDA approval, canine acceptance between Rimadyl® and Carprieve® chewable tablets has not been directly evaluated. Therefore, the objective of this study was to evaluate and compare the acceptability of two liver-flavored carprofen products (Carprieve® and Rimadyl® 25 mg chewable tablets) in 37 healthy purpose-bred dogs using a 2 × 2 complete cross-over design.

## Results

### Study population demographics

Thirty-seven dogs, including 18 females and 19 males, were enrolled in this study. On average, dogs weighed 10.6 ± 1.7 kg (range = 7.9 to 13.6 kg) and were 1.7 ± 0.5 years of age (range = 1.0 to 2.5 years). All dogs remained healthy throughout the study period and specifically no signs of gastrointestinal upset were observed. Overall, 37 individual acceptability tests were completed for each chewable carprofen tablet.

### Acceptance test

The study was initiated and completed over the same seven day period (September 20 to 27, 2018) for all dogs. Study population characteristics, carprofen tablet size administered, carprofen dose administered, and group allocation for all dogs are presented in Table 1. On study day 0, 19 and 18 dogs were offered Rimadyl® (Group II) or Carprieve® (Group I), respectively. After the seven-day “wash-out” period, 18 dogs were offered Rimadyl® (Group I) and 19 dogs were offered Carprieve® (Group II). Individual acceptability outcomes for days 0 and 7 for each dog are presented in Table 2. On study day 0, 67.6% (25/37) of dogs fully consumed the carprofen tablet offered, either Rimadyl® or Carprieve®, whereas 32.4% (12/37) of dogs did not accept either product (Table 3). Similarly, on study day 7, 75.7% (28/37) of dogs fully consumed the carprofen tablet and 24.3% (9/37) of dogs did not accept either product (Table 3). The majority of dogs fully consumed Rimadyl® (73.0%, 27/37) and Carprieve® (70.3%, 26/37) tablets, whereas 27.0% (10/37) and 29.7% (11/37) dogs did not accept Rimadyl® and Carprieve®, respectively (Table 4). The McNemar’s χ^2^ test indicated that acceptability did not significantly differ between Carprieve® and Rimadyl® carprofen tablets (McNemar’s χ^2^*P* = 0.65; Fisher exact test *P* = 1.00) (Table 5). Although not significantly different (*P* = 1.00), dogs offered Rimadyl® were 1.5 times (OR = 1.50; OR 95% confidence interval = 0.17–17.96) more likely to accept the tablet than dogs offered Carprieve®.

**Table 1:** Demographic characteristics, carprofen dose administered, and group allocation of dogs in the study

Group^*^	Dog ID	Sex^±^	Age, years	Weight, kg	Carprofen, mg^҂^	Dose, mg/kg
I	315–974	Female	1.0	10.4	25.0	2.4
I	439–468	Male	2.1	13.0	25.0	1.9
I	439–470	Male	1.2	11.6	25.0	2.2
I	440–118	Male	2.1	7.9	12.5	1.6
I	452–270	Female	1.5	8.1	12.5	1.5
I	540–556	Female	2.1	11.7	25.0	2.1
I	597–230	Male	2.0	12.7	25.0	2.0
I	597–674	Male	2.0	10.9	25.0	2.3
I	597–892	Male	1.1	11.3	25.0	2.2
I	600–010	Female	2.0	9.8	12.5	1.3
I	600–014	Female	2.0	9.4	12.5	1.3
I	600–236	Male	2.1	9.3	12.5	1.3
I	600–344	Male	2.0	9.8	12.5	1.3
I	600–816	Female	1.5	9.7	12.5	1.3
I	600–836	Male	1.0	13.3	25.0	1.9
I	600–934	Female	2.1	8.6	12.5	1.5
I	601–472	Female	2.1	8.4	12.5	1.5
I	601–663	Male	1.8	13.0	25.0	1.9
II	312–683	Male	1.0	11.4	25.0	2.2
II	312–987	Male	1.0	11.5	25.0	2.2
II	323–648	Female	2.5	9.3	12.5	1.3
II	439–977	Male	1.0	13.0	25.0	1.9
II	440–023	Female	2.0	11.9	25.0	2.1
II	440–104	Female	1.5	8.9	12.5	1.4
II	453–072	Male	1.0	13.6	25.0	1.8
II	597–303	Female	2.1	10.1	12.5	1.2
II	597–340	Female	1.6	9.4	12.5	1.3
II	597–362	Female	1.6	8.5	12.5	1.5
II	600–104	Male	2.1	10.3	25.0	2.4
II	600–324	Female	2.1	8.6	12.5	1.5
II	600–454	Male	1.0	12.7	25.0	2.0
II	600–779	Female	1.6	8.4	12.5	1.5
II	600–904	Male	2.1	9.9	12.5	1.3
II	601–341	Male	2.0	11.0	25.0	2.3
II	601–482	Female	2.0	10.7	25.0	2.3
II	601–928	Male	1.2	13.0	25.0	1.9
II	603–754	Female	2.1	10.3	25.0	2.4

^*^Group I was offered Carprieve® on day 0 and Rimadyl®; Group II was offered Rimadyl® on day 0 and Carprieve® on day 7

^±^All dogs were unaltered (i.e., sexually intact)

^**҂**^25.0 mg indicates a whole tablet was offered, 12.5 mg indicates a half tablet was offered

**Table 2:** Acceptability testing results for individual dogs on days 0 and 7

Dog ID	Day 0		Day 7	
	Treatment	Acceptability	Treatment	Acceptability
315–974	Carprieve	Full	Rimadyl	Full
439–468	Carprieve	Partial/none	Rimadyl	Full
439–470	Carprieve	Partial/none	Rimadyl	Partial/none
440–118	Carprieve	Partial/none	Rimadyl	Full
452–270	Carprieve	Full	Rimadyl	Full
540–556	Carprieve	Partial/none	Rimadyl	Full
597–230	Carprieve	Partial/none	Rimadyl	Partial/none
597–674	Carprieve	Partial/none	Rimadyl	Partial/none
597–892	Carprieve	Full	Rimadyl	Full
600–010	Carprieve	Full	Rimadyl	Full
600–014	Carprieve	Full	Rimadyl	Full
600–236	Carprieve	Full	Rimadyl	Partial/none
600–344	Carprieve	Full	Rimadyl	Full
600–816	Carprieve	Full	Rimadyl	Full
600–836	Carprieve	Full	Rimadyl	Full
600–934	Carprieve	Partial/none	Rimadyl	Partial/none
601–472	Carprieve	Full	Rimadyl	Full
601–663	Carprieve	Partial/none	Rimadyl	Partial/none
312–683	Rimadyl	Full	Carprieve	Full
312–987	Rimadyl	Partial/none	Carprieve	Full
323–648	Rimadyl	Partial/none	Carprieve	Partial/none
439–977	Rimadyl	Full	Carprieve	Full
440–023	Rimadyl	Full	Carprieve	Full
440–104	Rimadyl	Full	Carprieve	Full
453–072	Rimadyl	Full	Carprieve	Full
597–303	Rimadyl	Full	Carprieve	Full
597–340	Rimadyl	Full	Carprieve	Full
597–362	Rimadyl	Full	Carprieve	Full
600–104	Rimadyl	Partial/none	Carprieve	Partial/none
600–324	Rimadyl	Full	Carprieve	Full
600–454	Rimadyl	Full	Carprieve	Full
600–779	Rimadyl	Full	Carprieve	Full
600–904	Rimadyl	Full	Carprieve	Full
601–341	Rimadyl	Full	Carprieve	Full
601–482	Rimadyl	Full	Carprieve	Full
601–928	Rimadyl	Partial/none	Carprieve	Partial/none
603–754	Rimadyl	Full	Carprieve	Full

**Table 3:** Results of acceptability testing: Number of dogs fully or partially (or not) accepting either tablet by study day

Study day	Acceptability Outcome		Total
	Partial/none	Full	
0	12	25	37
7	9	28	37
Total	21	53	74

**Table 4:** Results of acceptability testing: Number of dogs fully or partially (or not) accepting a tablet by product

Treatment	Acceptability Outcome		Total
	Partial/none	Full	
Rimadyl®	10	27	37
Carprieve®	11	26	37
Total	21	53	74

**Table 5:** Results of acceptability testing: Paired analysis of acceptability results by product

Carprieve®	Rimadyl®		Total
	Full	Partial/none	
Full	24	2	26
Partial/none	3	8	11
Total	27	10	37

## Discussion

In this study, we demonstrated that canine acceptance did not significantly differ between Rimadyl® and Carprieve® carprofen chewable tablets when a single-dose was administered to healthy purpose-bred dogs in a 2 × 2 cross-over design. Palatability testing of orally administered veterinary pharmaceuticals is at the forefront of product development and marketing. There are two steps included within palatability testing, acceptance and preference testing. Acceptance testing, which is the most important measure of palatability in veterinary pharmaceuticals is designed to assess voluntary intake and, subsequently, offers a measure of compliance to the treatment protocol by the pet owner [12]. Currently, there are no standardized methods for acceptability testing of veterinary pharmaceuticals; consequently, palatability studies are largely based on principles outlined by the pet food industry [12, 16, 17]. Preference testing evaluates if the animal prefers one product over another.

Cross-over designs are preferred in acceptability testing to optimize sample size and allow for an unbiased evaluation of multiple formulations using the same individual. Canine palatability, acceptance and/or preference, of carprofen chewable tablets has been evaluated using cross-over designs previously; with all studies involving Rimadyl® compared to other carprofen formulations of various presentations, such as chewable tablet, caplet, or tablet [18–20]. In previous acceptability studies conducted in cross-over design, dogs were administered treatment on consecutive days [18–20]. Although not necessary to evaluate acceptability, and not included in other similar acceptability studies [18–20], a seven-day “wash-out” period was included in the present study to minimize the chance of conditioning the dogs to administration of the tablet, a presumed treat, so negative or favorable experiences did not interfere with observing the true acceptability of each tablet individually. Therefore, due to our study population and design limitations, we did not assess acceptability over a multiple day dosing regimen as would be typical for long-term OA treatment in pets. To evaluate long-term acceptability outcomes typical of pets treated for OA for Carprieve® and Rimadyl® chewable tablets, future research is warranted.

In one study, Rimadyl® was compared to two other carprofen products, Carprodyl® tablets (Ceva Animal Health; Amersham, United Kingdom) and Carprieve® caplets (formerly known as Norocarp® caplets), using acceptance and preference tests [18]. Following a complete cross-over design, 43 mixed breed dogs, aged between one to ten years old and weighing at least 10 kg, were randomly administered a carprofen tablet over two consecutive days [18]. Payne-Johnson et al.*,* found that of 43 dogs, 90.7 and 48.8% voluntary accepted Rimadyl® chewable tablets and Carprieve® caplets, respectively [18]. In this comparison between Rimadyl® chewable tablets and Carprieve® caplets, the acceptance tests were conducted using 75 mg and 50 mg carprofen formulations, respectively [18]. It has been documented that the concentration of active ingredient in the formulation, in this case—carprofen—can influence palatability [17]. While significant differences in acceptability and preference were observed in the previous study between Rimadyl® chewable tablets and Carprieve® caplets (*P* < 0.005), based on the product presentations compared, chewable tablets versus caplets, is not surprising [18]. In our study, we compared formulations of the same chewable tablet presentation formulated at 25 mg. Canine acceptance of Rimadyl® chewable tablets and Carprieve® chewable tablets was 73.0 and 70.3%, respectively. The chewable tablets in this study were formulated at 25 mg per tablet, however, the dose administered for acceptability testing was less than the recommended daily dose of 4.4 mg per kg of body weight (dosage administered ranged from 1.2 mg/kg to 2.4 mg/kg) but was approximate to the labeled halved daily dose of 2.2 mg/kg. Due to animal welfare concerns, given that our study subjects were healthy, we elected to not administer a complete target dose of carprofen, consistent with other carprofen acceptability studies in healthy dogs [18–20]. Thus, acceptability data should be interpreted with caution in the event where multiple chewable tablets would need to be given as treatment, as this study only administered half or whole tablets which may be more indicative of a dose given to a smaller dog.

In the present study, the study population of 37 dogs was very homogeneous in terms of age (1.7 ± 0.5 years of age) and breed (cross-bred Beagles) thus minimizing variability between dogs. Although this colony was readily available and purpose-bred for research, it has been documented, although anecdotally, that Beagles are a poor choice for use in palatability, namely preference studies; however, other extraneous factors such as inadequate acclimatization, laboratory versus in-home settings, and cultural differences such as use of treat rewards may outweigh any breed influence on palatability testing outcomes [12]. This study population may not be representative of typical pets or the target population of dogs experiencing a painful condition due to surgery or OA but it does offer an unbiased estimate of acceptability of these two products. Dogs suffering from OA, or recovering from surgery, may have a loss in appetite due to pain and stress which may ultimately impact acceptability compared to healthy, pain-free dogs [12]. Previous research (Norbrook Laboratories Limited, unpublished internal data) evaluated acceptability between Carprieve® and Rimadyl® 50 mg carprofen chewable tablets in 103 pet dogs with clinical symptoms requiring treatment by NSAIDs (e.g., hip dysplasia, spinal pain, OA). Acceptability was assessed after a single administration and no difference in acceptability was observed as 71.7 and 68.0% of dogs fully accepted Carprieve® and Rimadyl® chewable tablets, respectively (Norbrook Laboratories Limited, unpublished internal data). Whereas these findings are comparable to our present study in healthy purpose-bred dogs, these chewable tablets were formulated at a higher dose (50 mg) and were offered to dogs experiencing a painful condition.

Carprieve® chewable tablets are an FDA approved bioequivalent product to Rimadyl® chewable tablets; therefore, Carprieve® has demonstrated analogous pharmacokinetic properties, in addition to satisfactory safety and efficacy compared to Rimadyl®. A survey conducted by PetCareRx.com representing 1100 pet owners from 440 households noted the impact of pet healthcare costs influencing veterinary care and treatment [13]. The current study provides evidence that acceptability to Carprieve® chewable tablets did not differ from Rimadyl® chewable tablets; however, Carprieve®, as a generic, is generally marketed at a price point below that of Rimadyl® [21]. Although the majority of pet owners (82%) admit they would consider paying almost any amount of money to keep their pets healthy, 21% of dog owners said they have scaled back on veterinary visits due to costs [13]. Additional findings reported that 20% of owners take cost-cutting measures in terms of veterinary prescribed medications by purposely under-dosing the pet or by delaying or refusing purchasing the medication altogether to save money. Annually, it is estimated that pet owners spend on average $611 per pet, and $935 when pets have a chronic condition [13]. If orally administered veterinary pharmaceuticals are palatable, easy to administer, and affordable, pet owners will be more likely to provide the necessary medication to their pets as prescribed ultimately improving the dogs and owner’s quality of life.

## Conclusions

In this 2 × 2 complete cross-over experimental study including 37 healthy cross-bred Beagles, canine acceptability did not significantly differ between Carprieve® and Rimadyl® chewable tablets. Since pet owner compliance is critical for successful NSAID treatment in dogswith clinical OA or for other NSAID-based treatments, future acceptability studies evaluating the long-term administration between these two products, as well as other flavored/chewable generic carprofen products or NSAID classes are warranted.

## Methods

### Study population and study design

The target study population consisted of healthy purpose-bred, cross-bred Beagle dogs at least one year of age; there were no restrictions on age, breed, weight, or sex (spayed female, neutered male, intact females and males. Dogs were single sourced from an internal research colony housed at the study facility for approximately 12 months prior to study initiation where they participated in other unrelated, non-terminal research studies. All dogs were uniquely identified via microchip technology. Prior to study enrollment, all dogs were physically examined by the attending veterinarian and a chemistry panel to screen for liver and/or kidney abnormalities was performed. Dogs were randomly assigned to Group I or Group II, and thus administered two different types of carprofen chewable tablets for acceptance tests on day 0 and day 7. Randomization was performed using a random number generator in Microsoft® Excel® 2016 (Windows 10).

The study was designed as a 2 × 2 complete cross-over design (AB/BA design) where each dog was randomly offered Carprieve® or Rimadyl® on day 0 and, after a seven-day “wash-out” period, offered the alternate carprofen chewable tablet. All dogs were weighed prior to acceptability testing on study day − 1 to determine appropriate dose for testing. To avoid potential overdose and adverse events, the attending veterinarian recommended using the halved daily dose (2.2 mg/kg) as a single dose for this study. Further, it was determined that doses should be rounded to the nearest half or whole tablet, in this way the division of tablets would be minimized to no more than one division with the assumption being that the number of divisions could potentially confound the acceptance of the tablet. Adverse events associated with administration of carprofen per label include vomiting, diarrhea, changes in appetite, lethargy, behavioral changes, and constipation. Therefore, dogs were offered either a half (12.5 mg, bodyweight ≤10.2 kg) or whole carprofen chewable tablet (25.0 mg, bodyweight > 10.3 kg) according to their body weight on day 0. Dogs were not reweighed prior to study day 7 and were offered the same dosage for both brands of chewable tablets. General health observations were performed twice daily by animal care staff on all dogs for the seven-day study period. All dogs remained in the research colony following this study.

### Animal care and housing

This study was conducted as a non-good laboratory practice (non-GLP) study at the Veterinary and Biomedical Research Center, Inc. (VBRC, Inc.; Manhattan, KS), a GLP compliant and fully accredited Association for Assessment and Accreditation of Laboratory Animal Care (AAALAC) facility. The study protocol was internally reviewed and approved by the Norbrook Laboratories Limited Research and Development personnel. Additionally, the study protocol was submitted to the VBRC, Inc. Institution for Animal Care and Use Committee (IACUC) where the protocol received approval prior to study initiation.

Dogs were housed indoors, individually or paired with the same sex, in raised, stainless-steel kennels with access to a resting pad, water, food, and toys for enrichment. Indoor facilities were maintained according to AAALAC requirements, encompassing the Guide for the Care and Use of Laboratory Animals, with an ambient temperature of 10.0 °C to 26.7 °C and a 12:12 h light:dark light cycle throughout the study [14]. All dogs received human interaction, as one form of provided enrichment, at minimum, twice per day. A commercial dry-food diet was fed twice daily with at least 8 h between feedings, based on body weight; dogs housed in same-sex pairs were separated for acceptance testing and feedings. Water was provided ad libitum.

### Acceptance test

Acceptance testing was conducted approximately one hour prior to the morning feeding time outlined per testing facility site standard operating procedures. The carprofen products were stored in a padlocked safe ensuring the products kept dry, out of direct-sunlight, and were maintained at room temperature (20 °C to 25 °C). The product labels were covered with a handwritten label containing an “A” or “B” by an unmasked individual (KD) so the product could not be identified by study personnel. Prior to acceptability testing, the appropriate number of chewable tablets were halved by an unmasked individual (KD). The whole or half tablets were removed from their relabeled original container, using a pair of forceps with one pair dedicated to each brand by the unmasked individual (KD) and placed into the gloved right hand of the acceptability test administrator who was blinded to treatment (DV). Gloves were changed between each individual acceptance test to keep acceptability tests consistent and unbiased for all dogs with no potential for carryover of scent or taste from the previous test article or dog. Acceptance of the tablets was assessed separately for each individual dog by offering the carprofen chewable tablet (Rimadyl® or Carprieve®) in a clean bowl and giving the dog the opportunity to voluntarily prehend and ingest the tablet. The dogs were given 60 s, measured with the use of a handheld stopwatch, to ingest the tablet. If the tablet was not completely consumed after 60 s it was then offered by the right gloved-hand of the test administrator (DV) for an additional 60 s without encouragement or coercion to ingest the offered tablet. Testing was terminated if the dog did not voluntarily ingest the tablet in the two minutes allotted and the remaining tablet was disposed of. Acceptability outcomes were recorded as “full” or “partial/none”. Acceptability was recorded as “full” if the dog completely consumed the tablet offered from 1) the bowl within 60 s, and if not accepted from the bowl, 2) the right gloved-hand within 60 s. If the dog did not completely consume the offered tablet or did not prehend the tablet at all when offered in the bowl or by gloved-hand, the acceptability outcome was recorded as “partial/none”.

### Sample size determination

A total of 74 dogs, or 37 in cross-over design, were required to detect a difference of 15% or greater in acceptability between two products (Rimadyl® and Carprieve®) with a 95% (α = 0.05) certainty that the difference is real and not due to chance alone with a type II error rate of 20% (*β* = 0.80), as calculated using a predicted acceptability of 95% [15].

### Statistical analysis

Carprofen chewable tablet brands were coded prior to statistical analysis, hence the individual (DV) performing analysis was blinded to treatment groups. The individual dog was considered the experimental unit. The outcome of the acceptability test consisted of “full” or “partial/none” for each carprofen tablet for each dog. Descriptive statistics were summarized using two-way frequency tables presenting acceptability by study day, and by carprofen product. To account for the cross-over design, acceptability test results were matched by dog and classified into one of the four categories: 1) neither tablet accepted, 2) both tablets accepted, 3) only Carprieve® accepted, or 4) only Rimadyl® accepted. A McNemar’s Chi-squared (χ^2^) test was performed in Stata® 12.0 (StataCorp LP, College Station, Texas), using the calculated frequencies of the four categories previously described, accounting for the 1:1 paired data. Odds ratios and exact Fisher confidence intervals were obtained. Differences in acceptability were considered significant if McNemar’s χ^2^ *P* ≤ 0.05.

## Data Availability

All data generated and analyzed during the current study are included in this published article (Tables 1, 2 to 3).

## Abbreviations

FDA: Food and Drug Administration
NSAIDs: Non-steroidal anti-inflammatory drugs
OA: Osteoarthritis

## References

[1] Johnston SA (1997). Osteoarthritis, joint anatomy, physiology and pathobiology. Vet Clin North Am Small Anim Pract.

[2] Sanderson RO, Beata C, Flipo RM, Genevois JP, Macias C, Tacke S, Vezzoni A, Innes JF (2009). Systematic review of the management of canine osteoarthritis. Vet Rec..

[3] Cook JL, Payne JT (1997). Surgical treatment of osteoarthritis. Vet Clin North Am Small Anim Pract.

[4] Brainard BM, Meredith CP, Callan MB, Budsberg SC, Shofer FS, Driessen B, Otto CM (2007). Changes in platelet function, hemostasis, and prostaglandin expression after treatment with nonsteroidal anti-inflammatory drugs with various cyclooxygenase selectivities in dogs. Am J Vet Res.

[5] Lipscomb VJ, AliAbadi FS, Lees P, Pead MJ, Muir P (2002). Clinical efficacy and pharmacokinetics of carprofen in the treatment of dogs with osteoarthritis. Vet Rec.

[6] Mansa S, Palmér E, Grøndahl C, Lønaas L, Nyman G (2007). Long-term treatment with carprofen of 805 dogs with osteoarthritis. Vet Rec..

[7] Moreau M, Dupuis J, Bonneau NH, Desnoyers M (2003). Clinical evaluation of a nutraceutical, carprofen and meloxicam for the treatment of dogs with osteoarthritis. Vet Rec..

[8] Pollmeier M, Toulemonde C, Fleishman C, Hanson PD (2006). Clinical evaluation of firocoxib and carprofen for the treatment of dogs with osteoarthritis. Vet Rec..

[9] Vasseur PB, Johnson AL, Budsberg SC, Lincoln JD, Toombs JP, Whitehair JG, Lentz EL (1995). Randomized, controlled trial of the efficacy of carprofen, a nonsteroidal anti-inflammatory drug, in the treatment of osteoarthritis in dogs. J Am Vet Med Assoc.

[10] United States Food and Drug Administration: Get the facts about pain relievers for pets. https://www.fda.gov/AnimalVeterinary/ResourcesforYou/AnimalHealthLiteracy/ucm392732.htm#Approved (2018). [Accessed 17 Jan 2018].

[11] Talamonti Zita, Cassis Chiara, Brambilla Paola G., Scarpa Paola, Stefanello Damiano, Cannas Simona, Minero Michela, Palestrini Clara (2015). Preliminary Study of Pet Owner Adherence in Behaviour, Cardiology, Urology, and Oncology Fields. Veterinary Medicine International.

[12] Thombre AG (2004). Oral delivery of medications to companion animals: palatability considerations. Adv Drug Del Rev.

[13] DVM360.com: New study confirms pet owners’ concerns about veterinary care costs. http://veterinarynews.dvm360.com/print/402954?page=full [Accessed 07 Dec 2018].

[14] National Research Council: Guide for the care and use of laboratory animals, Eighth Edition. Available from: https://grants.nih.gov/grants/olaw/guide-for-the-care-and-use-of-laboratory-animals.pdf (2011) [Accessed 25 Jan 2019].

[15] Sealed Envelope Ltd: Power calculator for binary outcome equivalence trial. Available from: https://www.sealedenvelope.com/power/binary-equivalence/ (2012) [Accessed 21 Jan 2019].

[16] Aldrich GC, Koppel K (2015). Pet food palatability evaluation: a review of standard assay techniques and interpretation of results with a primary focus on limitations. Animals..

[17] Aleo M, Ross S, Becskei C, Coscarelli E, King V, Darling M, Lorenz J (2018). Palatability testing of oral chewables in veterinary medicine for dogs. Open J Vet Med.

[18] Payne-Johnson M, Maitland TP, Bullard J, Gossellin J (2006). Comparative palatability of three commercial formulations of carprofen and one commercial formulation of firocoxib in dogs. Revue Méd Vét..

[19] Payne-Johnson M, Maitland TP, Tilt N, Gossellin J (2007). An evaluation of the relative palatability of two commercial oral tablet formulations of carprofen and meloxicam in dogs using acceptance and preference tests. Revue Méd Vét.

[20] Gossellin J, Maitland TP, Civil J (2010). Relative preference of dogs for two commercial oral tablet formulations of carprofen. Revue Méd Vét.

[21] Valley Vet Supply: Carprofen chewable tablets. https://www.valleyvet.com/search.html?gas=carprofen%20chewable%20tablets [Accessed 16 Jan 2019].

